# The New York Quarantine Difficulty

**Published:** 1859-04

**Authors:** 


### *The New York Quarantine Difficulty.*

Dr. W. C. Anderson, President of the Richmond County (Staten Island) Medical Society, in a communication to the Commissioners of Emigration, presents the following propositions as a solution of the quarantine difficulties. "A floating hospital for yellow fever patients to be anchored at the Southwest Spit, all other patients to be sent to Ward's Island; and the occupation of the point of Sandy Hook by the general government for warehouses for the storage of infected cargoes—the anchorage of vessels supposed to be infected, to be for the future in the lower bay."

We are indebted to Dr. Anderson for the following description of the hospital ship Caledonia, the Marine Hospital for the port of London, England.

"Her principal dimensions are:—Length of gun deck, 200 feet; length of keel, for tonnage, 170 feet 9 inches; extreme breadth, 53 feet 8 inches; depth in hold, 23 feet 2 inches; burden in tons, 2,216. The admission and circulation of a healthy atmosphere have been studied with considerable care, for which end a large portion of each deck midships has been left open, and from the lower decks upwards, open hatches, guarded by iron rails, have been established. At the stern of the ship are likewise improved galleries connected with the respective docks, and easily accessible, adapted for the convenience of removing patients, when necessary, into the open air; the weather deck is fitted with a number of lantern lights, six feet high, and from its spacious accommodation, forms an admirable promenade for the patients. The forecastle is arranged as cabins for the petty officers of the ship and their families. The poop is occupied by the superintendent, solely. The main deck is laid out in the following manner:—The whole of the after part is adapted as a dining room and general quarters for the principal medical officer, surgeon, assistant surgeon and chaplain; the starboard side is separated by a gothic screen of old oak wood, and is converted into an elegant chapel, capable of containing sixty persons; the port side of this deck is hung with fifty or sixty hammocks, for the accommodation of convalescents, removed from the sick wards, while the fore part of the same deck serves as quartermasters' cabins, female nurses' dining room, carpenter's workshop, dispensary, boatswain's storeroom, etc.

"On the aft starboard side of the middle deck, in close proximity with an open gallery, is a commodious room for surgical operations; the other portions of this deck afford a space for the reception of cases of accident, and cabins for the nurses.

"The lower gundeck contains a museum, quarters for the fever patients, and cabins for the hospital attendants; a portion of the orlop deck is appropriated as a dead house, and is fitted up with a slate table for the purpose of post mortem examinations. This deck also contains the laboratories and hair cutting rooms. Two pumps are fitted in this deck, with an engine hose, in case of fire, serves for washing decks and various other uses. One of the ports has been enlarged four feet square, so as to admit patients in cases of emergency.

"The various apartments of the ship are heated from this deck, with supply pipes laid on each deck, with hot air. Here are also six tanks for containing a good supply of fresh water and stores, and for housing upward of six hundred tons of coal and coke. With this minute attention to the comfort and convenience of the officers and attendants, nothing has been neglected which could secure the well being of the patients, and the success which the medical and surgical department of the floating hospital realize is in a great measure due to the admirable arrangements which could not be so well accomplished on shore.

"In illustration of this fact, I can quote the testimony of the medical officers, to the effect that erysipelas and hospital gangrene, so fatal in the hospitals of London and Paris, are never the consequence of operating in the floating hospitals. This is a most important fact in point of superior facilities for ventilation and supply of fresh air, so necessary in the treatment of the sick, especially in cases of infectious diseases; and it also affords presumptive evidence of the best kind, that yellow fever might be treated on board the floating hospital without the danger that has been suggested by 'Commissioners for Quarantine removal,' of infecting the ship, and its being retained and propagated, ad infinitum, by bilge water."—*Medical & Surgical Reporter.*

